# Predictive factors for pleural drainage volume after uniportal video-assisted thoracic surgery lobectomy for non-small cell lung cancer: a single-institution retrospective study

**DOI:** 10.1186/s12957-020-01941-5

**Published:** 2020-07-08

**Authors:** Ming-bo Tang, Jia-lin Li, Su-yan Tian, Xin-liang Gao, Wei Liu

**Affiliations:** 1 Department of Thoracic Surgery, The First Hospital of Jilin University, 71 Xinmin Street, Changchun, Jilin, 130021 China; 2 Department of Division of Clinical Research, The First Hospital of Jilin University, Changchun, Jilin, 130021 China

**Keywords:** Predictive factors, Pleural drainage volume, Uniportal VATS lobectomy, Non-small cell lung cancer

## Abstract

**Objective:**

To identify the predictive factors associated with pleural drainage volume (PDV) after uniportal video-assisted thoracic surgery (VATS) lobectomy for non-small cell lung cancer (NSCLC).

**Methods:**

A total of 440 consecutive NSCLC patients who underwent uniportal VATS lobectomy were enrolled in this study between November 2016 and July 2019. Thirty-four parameters, including patients’ clinicopathological characteristics and other potential predictors were collected. Daily drainage volume was summed up as PDV. Univariate analysis and multivariate regression models were fitted to identify independent predictive factors for PDV.

**Results:**

The median PDV was 840 ml during the median drainage duration of 4 days. A strong correlation was observed between PDV and drainage duration (correlation coefficient = 0.936). On univariate analysis, age, forced expiratory volume in 1 s % predicted (FEV1%), left ventricular ejection fraction (LVEF), operation time, serum total protein (TP), and body mass index (BMI) showed a significant correlation with PDV (*P* value, **<** 0.001, **<** 0.001, 0.003, 0.008, 0.028, and 0.045, respectively). Patients with smoking history (*P* = 0.030) or who underwent lower lobectomy (*P* = 0.015) showed significantly increased PDV than never smokers or those who underwent upper or middle lobectomy, respectively. On multivariate regression analysis, older age (*P***<** 0.001), lower FEV1% (*P***<** 0.001), lower LVEF (*P* = 0.011), lower TP (*P* = 0.013), and lower lobectomy (*P* = 0.016) were independent predictors of increased PDV.

**Conclusions:**

Predictive factors of PDV can be identified. Based on these predictors, patients can be treated with tailored individualized safe chest tube management.

## Introduction

Postoperative chest tube placement to drain pleural fluid is an important procedure after thoracic surgery. Pleural drainage volume (PDV) after the operation is directly related to the duration of drainage. A higher PDV will lead to prolonged placement of the chest tube and result in an uncomfortable experience of pain, immobilization, increased risk of infection [1, 2], as well as increased length and cost of hospitalization subsequently [3]. Besides, very early removal of the chest tube in patients with excessive PDV might also cause frequent chest tube replacement because of symptomatic effusion [4].

Thus, the timing of chest tube removal has always been a controversial issue. Empirically, the threshold of daily drainage volume depends on the experience of different surgeons [5]. This threshold varies widely; it can be 2 ml/kg body weight [6], or it can be 100 ml [7], 200 ml [8], or even 500 ml per day [9]. Occasionally, the daily drainage volume is not considered during chest tube removal [10, 11]. However, the uniform standard may not be the appropriate threshold level for different patients as the ability of the pleura for filtration and reabsorption vastly differs from patient to patient. Under this circumstance, a thoracic surgeon should be able to accurately predict the drainage volume for appropriate chest tube management.

To the best of our knowledge, no study has systematically investigated the predictive factors for total PDV after lobectomy. To address this issue, we retrieved the daily drainage records of non-small cell lung cancer (NSCLC) patients who underwent uniportal video-assisted thoracic surgery (VATS) lobectomy and we extensively analyzed the potential predictive factors associated with PDV.

## Methods

### Patients

**Fig. 1:**
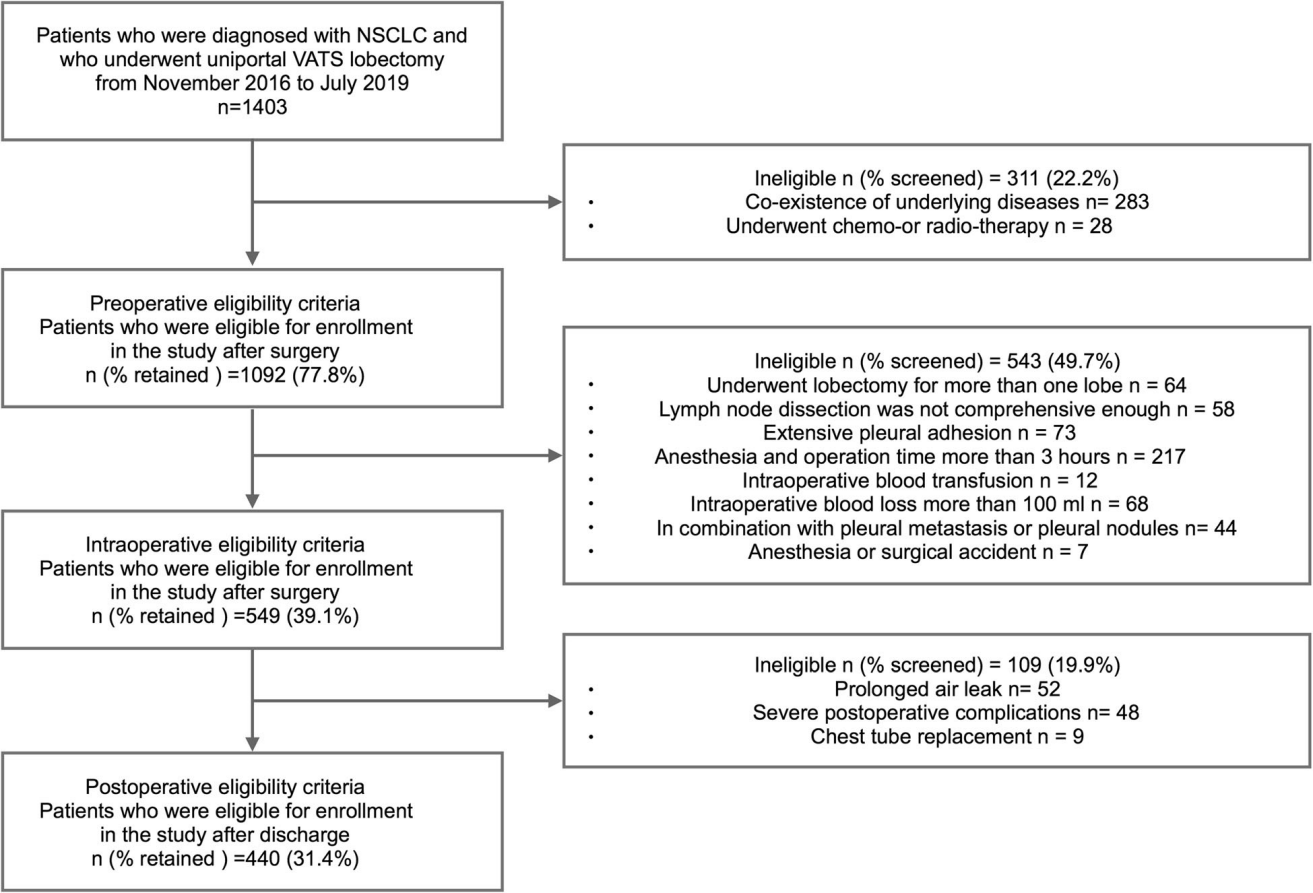
Flowchart for patient screening

The present study enrolled 440 consecutive NSCLC patients who underwent uniportal VATS lobectomy and systematic lymph node dissection between November 2016 and July 2019. All patients were screened according to the following exclusion criteria: co-existence of severe underlying diseases before the operation; in combination with pleural metastasis or pleural nodules; lobectomy for more than one lobe; chemo- or radio-therapy prior to surgery; anesthesia and operation time more than 3 h; detection of extensive pleural adhesion intraoperatively; intraoperative blood transfusion; intraoperative blood loss more than 100 ml; anesthesia or surgical accident; severe postoperative complications; prolonged air leak; and chest tube replacement. The flow chart for selection of patients is shown in Fig. 1. The tumor, node, metastasis (TNM) staging was in accordance with the International Association for the Study of Lung Cancer (IASLC) staging system (the 8th Edition) [12]. This study was approved by the Ethics Committee of First Hospital of Jilin University. Informed consent for use of the medical data in this study was obtained from the patients.

### Perioperative management

All patients underwent routine preoperative examination and blood tests in our hospital. The enrolled patients underwent uniportal VATS lobectomy through an incision 4–5 cm long over the fifth intercostal space under single lung ventilation. The pulmonary vessels and bronchi were individually dissected, stapled, or ligated and then they were divided. Incomplete fissures were developed by mechanical staplers. Lobectomy and systematic lymph node dissection were performed in all enrolled patients. Mediastinal lymph nodes were completely resected at stations 4L, 5, 6, 7, 8, and 9 for left-sided cancers; and at stations 2R, 4R, 7, 8, and 9 for right-sided cancers. After confirmation of achievement of pneumostasis and hemostasis, a chest tube was conventionally placed through the incision over the fifth intercostal space apically before chest closure. The tube was connected to a drainage bucket with a negative pressure of 8 cm H_2_O suction during the first 2 days after surgery. Routine chest X-ray was performed on the first day after the operation, and it was reperformed before chest tube removal.

The total PDV was calculated based on the records obtained by a particular doctor every 24 h. Postoperative management mainly focused on early ambulation, antibiotic prophylaxis, respiratory rehabilitation, and anticoagulation. The chest tube was removed once the following conditions were fulfilled: chest X-ray showed satisfactory lung re-expansion; there was absence of air leak in the chest drainage bucket; there was absence of thick bloody, purulent, or cloudy pleural fluid; and the drainage volume was not more than 200 ml on the day of chest tube removal.

### Statistical data analysis

A total of 34 parameters of patients were analyzed in this study. Continuous variables were summarized as mean ± standard deviation for the normally distributed data and as median (interquartile range [IQR]) for the non-normally distributed data. In univariate analysis of a continuous variable, Spearman correlation coefficients were calculated between PDV and the continuous variable. For a categorical variable, Mann–Whitney *U* tests were performed for comparison between two groups and Kruskal–Wallis test was performed for comparison among three or more groups. Variables with a *P* value < 0.1 on univariate analysis were entered into multivariate regression analysis with backward selection to identify independent predictors of PDV, wherein categorical variables were converted into dummy variables, such as 0 or 1 for patients with or without smoking history. Values of *P* < 0.05 were considered statistically significant. All analyses were performed with Statistical Package for Social Sciences (SPSS) version 23.0 (SPSS Inc., Chicago, IL, USA). Graphs were created with the aid of the GraphPad Prism version 8.2.0.

## Results

**Fig. 2:**
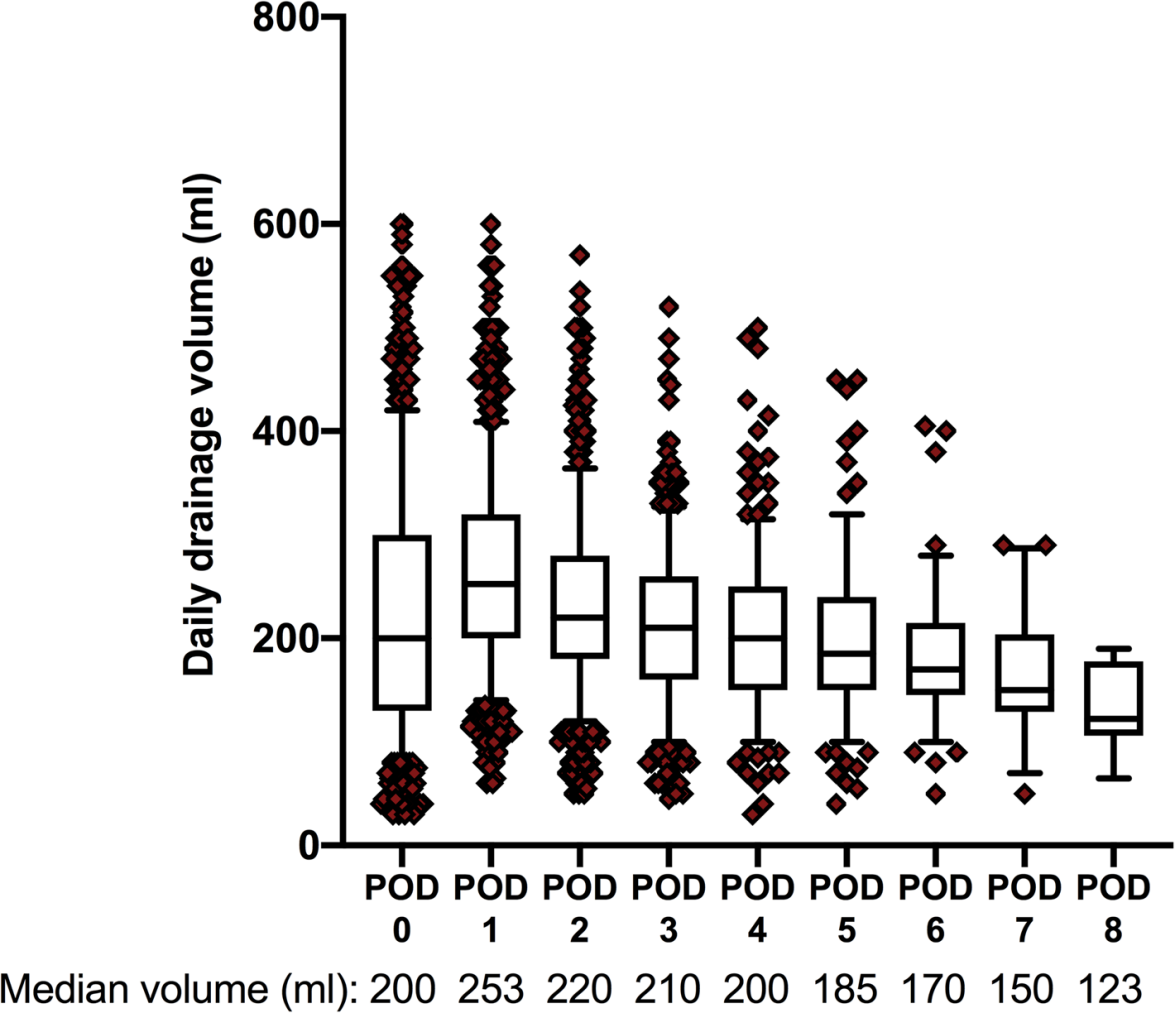
Daily drainage volume is shown in the form of a graph. The box plots represent IQRs. The lower and upper bars show the 10th percentile and the 90th percentile, respectively; the circles represent the outliers. The median drainage volumes on each POD are also presented. POD postoperative day, IQR interquartile range

The total 440 patients included 268 women and 172 men, and the median age was 60 years. The median PDV was 840 ml during the median drainage duration of 4 days. Further details of the clinicopathologic characteristics are shown in Table 1. The volume of daily drainage on the particular postoperative day (POD) is shown in Fig. 2, and it showed a general decreasing trend from the first day after the operation. A strong correlation was observed between PDV and duration of drainage (correlation coefficient, 0.936; *P***<** 0.001).

**Table 1:** Clinicopathological characteristics of the enrolled patients

Parameters	
Age (years, IQR)	60 (53–65)
Gender male (*n*, %)	172 (39.1%)
Smoking history (*n*, %)	130 (29.5%)
TNM staging (*n*, %)	
IA	241 (54.8%)
IB	99 (22.5%)
IIA	16 (3.6%)
IIB	53 (12.0%)
IIIA	21 (4.8%)
IIIB	5 (1.1%)
IV	5 (1.1%)
Pathology (*n*, %)	
Adenocarcinoma	385 (87.5%)
Squamous carcinoma	46 (10.5%)
Others	9 (2.0%)
Surgical location (*n*, %)	
LUL	104 (23.6%)
LLL	79 (18%)
RUL	121 (27.5%)
RML	39 (8.9%)
RLL	97 (22%)
Duration of drainage (days, IQR)	4 (3–5)
PDV (ml, IQR)	840 (570–1370)

*IQR* interquartile range, *TNM* tumor, node, metastasis, *LUL* left upper lobe, *LLL* left lower lobe, *RUL* right upper lobe, *RML* right middle lobe, *RLL* right lower lobe

**Fig. 3:**
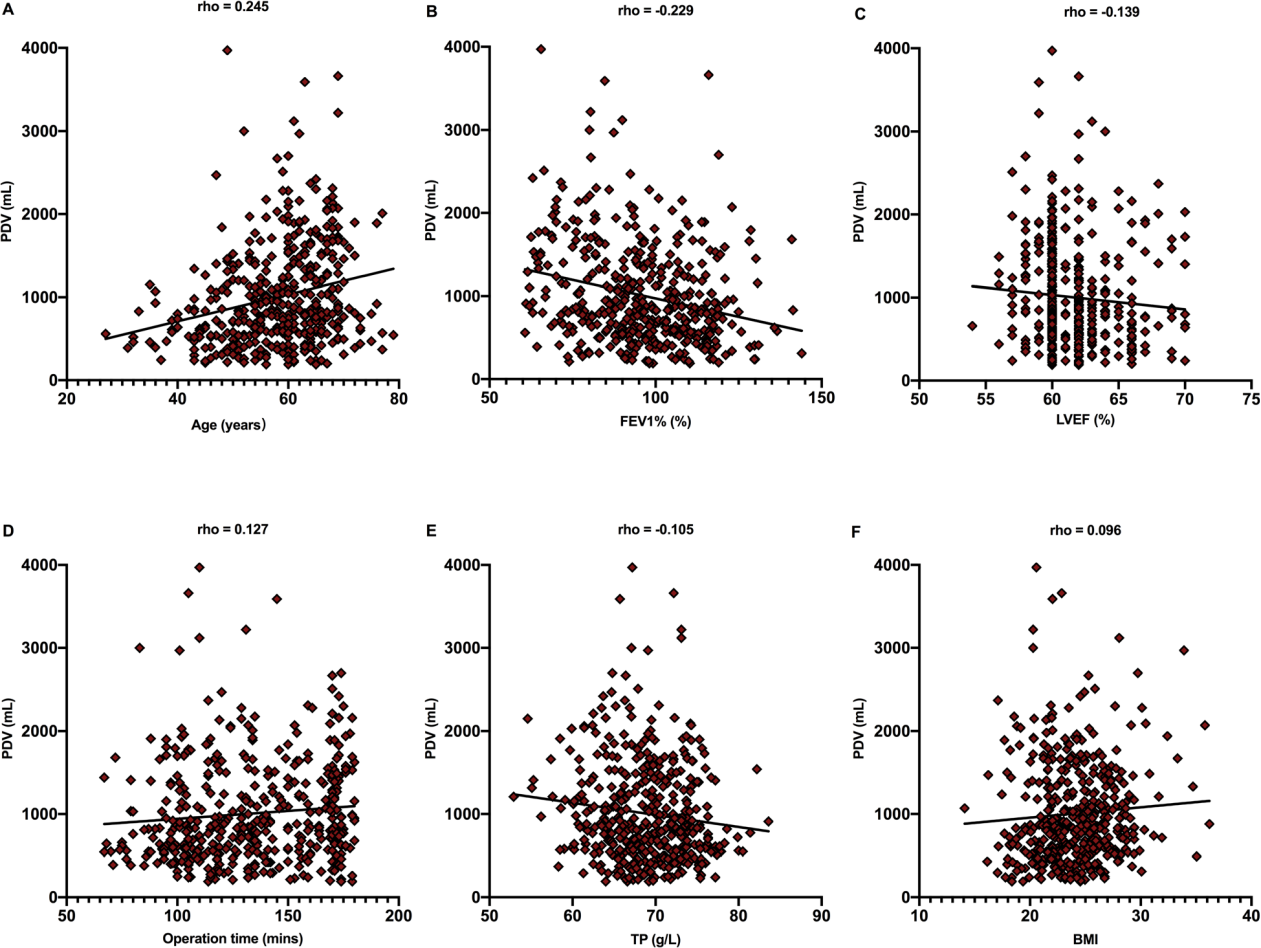
Correlations between PDV and each factor with statistical significance. **a** Age, **b** FEV1%, **c** LVEF, **d** Operation time, **e** TP, **f** BMI. PDV pleural drainage volume, FEV1% forced expiratory volume in 1 s % predicted, LVEF left ventricular ejection fraction, TP serum total protein BMI body mass index

Univariate correlation analysis showed that age, operation time, and BMI were positively correlated with PDV (*P* value, **<** 0.001, 0.008, and 0.045, respectively). In contrast, FEV1%, LVEF, and TP showed a negative correlation with PDV (*P* value, **<** 0.001, 0.003, and 0.028, respectively). The correlations between PDV and each factor with statistical significance are shown in Fig. 3. After using a less stringent cut-off of 0.1 for *P* values, statistically significant factors, such as left ventricular fractional shortening (LVFS), serum albumin (ALB), activated partial thromboplastin time (APTT), white blood cell count (WBC), carbon monoxide lung diffusion capacity % predicted (DLCO%), serum creatinine (CRE), prothrombin time (PT), and international normalized ratio (INR) were included in the multiple regression analysis (*P* value 0.059, 0.076, 0.063, 0.056, 0.063, 0.081, 0.089, and 0.091, respectively; Table 2). The correlations between PDV and other parameters were not statistically significant, including aspartate aminotransferase (AST), alanine aminotransferase (ALT), blood urea nitrogen (BUN), thrombin time (TT), fibrinogen (FIB), red blood cell count (RBC), platelet count (PLT), hemoglobin (Hb), blood glucose, and erythrocyte sedimentation rate (ESR).

**Table 2:** The correlation between PDV and factors with *P* < 0.1 from continuous variables

Factors	Mean ± SD or median (IQR)	Correlation coefficient	*P* value
Age (years)	60 (53–65)	0.245	***< 0.001***
BMI (kg/m^2^)	24.01 ± 3.34	0.096	***0.045***
Operation time (min)	132 (108–164)	0.127	***0.008***
FEV1% (%)	96.2 ± 16.7	− 0.229	***< 0.001***
DLCO% (%)	89.1 ± 16.8	− 0.089	0.063
LVFS (%)	32.5 ± 2.3	− 0.090	0.059
LVEF (%)	61.5 ± 2.8	− 0.139	***0.003***
TP (g/l)	68.85 ± 4.92	− 0.105	***0.028***
ALB (g/l)	40.63 ± 3.44	− 0.085	0.076
CRE (μmol/l)	64.20 ± 13.67	0.083	0.081
APTT (s)	27.32 ± 3.36	0.089	0.063
PT (s)	10.75 ± 0.83	0.081	0.089
INR	0.96 ± 0.06	0.081	0.091
WBC (10^9^/l)	5.87 ± 1.49	0.091	0.056

*PDV* pleural drainage volume, *IQR* interquartile range, *BMI* body mass index, *FEV1%* forced expiratory volume in 1 s % predicted, *DLCO%* carbon monoxide lung diffusion capacity % predicted, *LVFS* left ventricular fractional shortening, *LVEF* left ventricular ejection fraction, *TP* serum total protein, *ALB* serum albumin, *CRE* serum creatinine, *PT* prothrombin time, *APTT* activated partial thromboplastin time, *INR* international normalized ratio, *WBC* white blood cell count

**Fig. 4:**
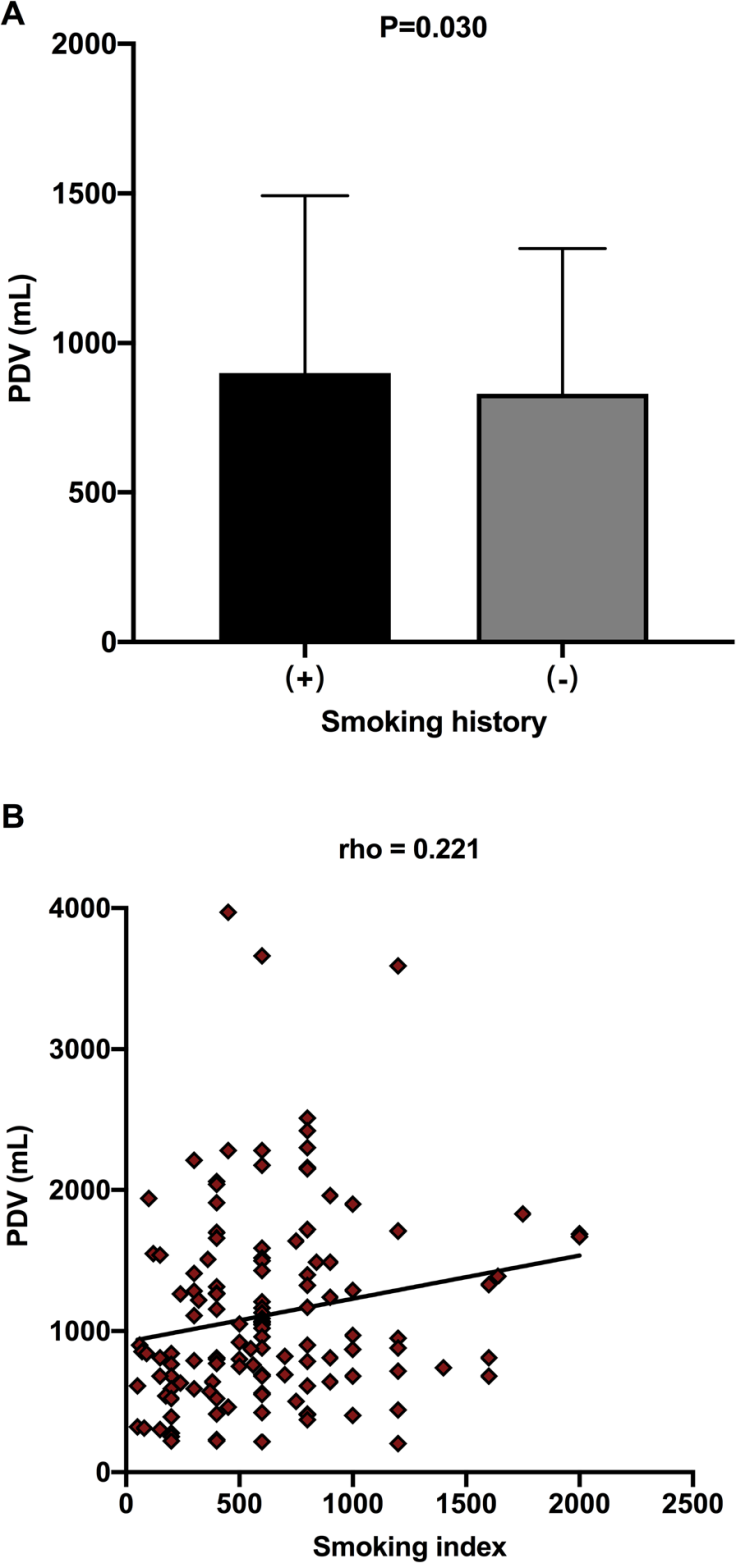
The comparison of PDV between patients with and without smoking history, and the correlation between the smoking index and PDV in patients with smoking history. **a** The median PDV of patients with smoking history was higher than that of never smokers (900 ml vs. 830 ml; *P* = 0.030). **b** The smoking index showed a positive correlation with PDV in patients with smoking history, and the correlation coefficient (rho) was 0.221. PDV pleural drainage volume

**Fig. 5:**
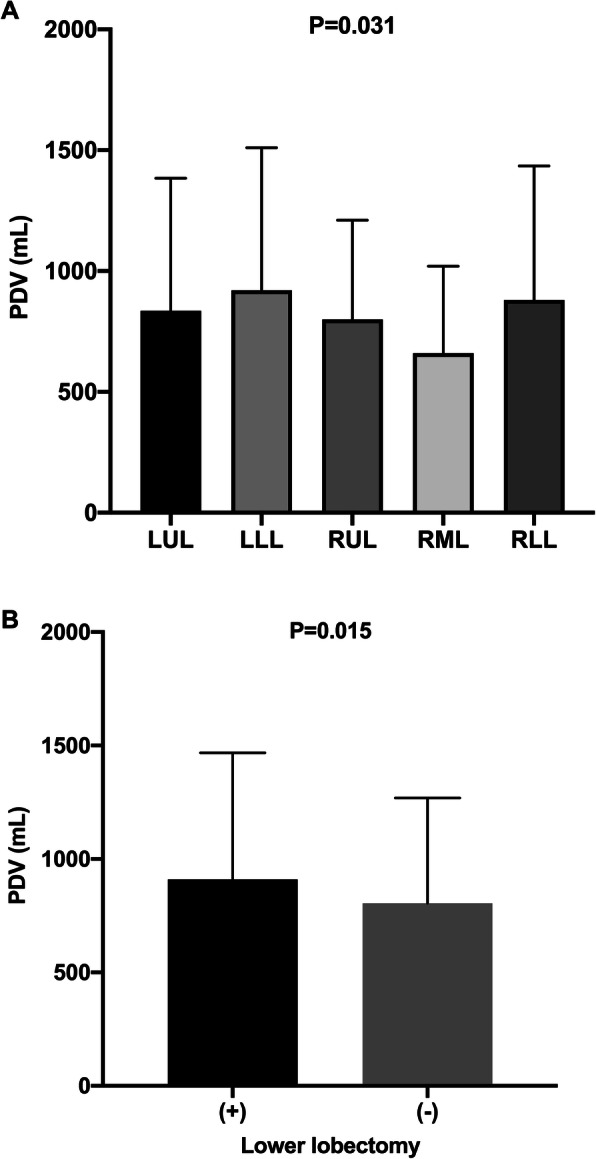
PDV in patients with each type of lobectomy and the comparison between lower lobectomy and upper lobectomy. **a** The median PDV of patients who underwent LUL, LLL, RUL, RML, and RLL was 835, 920, 800, 660, and 880 ml, respectively (*P* = 0.031). **b** Patients who underwent lower lobectomy showed a higher PDV compared with those who underwent upper lobectomy (910 ml vs. 805 ml; *P* = 0.015). PDV pleural drainage volume, LUL left upper lobe, LLL left lower lobe, RUL right upper lobe, RML right middle lobe, RLL right lower lobe

Patients with smoking history showed a significantly higher PDV compared with never smokers (900 ml vs. 830 ml; *P* = 0.030; Table 3; Fig. 4a). Among the patients with smoking history, the smoking index showed a positive correlation with PDV (*P* = 0.011; Fig. 4b). There was no statistically significant difference in PDV between male and female patients (*P* = 0.167). Among PDVs after different types of lobectomies, PDV after left lower lobectomy was the highest, while PDV after right middle lobectomy was the lowest. Overall, the PDV levels among patients with different types of lobectomy differed substantially (*P* = 0.031; Table 3; Fig. 5a). Patients who underwent lower lobectomy had a significantly higher PDV of 910 ml compared to patients who underwent upper or middle lobectomy and showed a PDV of 805 ml (*P* = 0.015; Table 3; Fig. 5b). No significant difference in PDV was observed in patients who underwent lobectomy on the left side or the right side (*P* = 0.152). Although PDV slightly increased by the stage of the tumor, the difference was not statistically significant. With respect to the T-stage, the PDVs of T1, T2, T3, and T4 stage patients were 810, 865, 972.5, and 1020 ml, respectively (*P* = 0.119). With respect to the N-stage, PDVs of N0, N1, and N2 stage patients were 830, 990, and 1045 ml, respectively (*P* = 0.239). With respect to TNM staging, PDVs of stage I, II, III, and IV patients were 830, 882.5, 955, and 950 ml, respectively (*P* = 0.471). In addition, patients with tumor invasion of the pleura had a higher PDV (910 ml vs. 820 ml, *P* = 0.122), and the pathological subtype of patients did not significantly affect the PDV (*P* = 0.765).

**Table 3:** Comparison of PDV based on factors with *P* < 0.1 from categorical variables

Factors	PDV (ml, IQR)	*P* value
Smoking history		***0.030***
With	900 (640–1493)	
Without	830 (550−1315)	
Surgical location		***0.031***
LUL	835 (552.5−1384)	
LLL	920 (610−1510)	
RUL	800 (525−1210)	
RML	660 (430−1020)	
RLL	880 (630−1435)	
Surgical location		***0.015***
Upper or middle lobe	805 (540−1269)	
Lower lobe	910 (630−1468)	

*PDV* pleural drainage volume, *IQR* interquartile range, *LUL* left upper lobe, *LLL* left lower lobe, *RUL* right upper lobe, *RML* right middle lobe, *RLL* right lower lobe

Using the multiple backward regression strategy, age (*P***<** 0.001), FEV1% (*P***<** 0.001), LVEF (*P* = 0.011), TP (*P* = 0.013), and lower lobectomy (*P* = 0.016) were included in the final model. Specifically, older age, lower FEV1%, lower TP, lower lobectomy, and lower LVEF were associated with an increase in the total PDV after uniportal VATS lobectomy in NSCLC patients (Table 4).

**Table 4:** Multivariate regression analysis of PDV

Factors	Regression coefficients	*P* value
Age	0.203	***< 0.001***
FEV1%	− 0.225	***< 0.001***
LVEF	− 0.258	***0.011***
TP	− 0.112	***0.013***
Lower lobectomy	0.109	***0.016***

*PDV* pleural drainage volume, *FEV1%* forced expiratory volume in 1 s % predicted, *LVEF* left ventricular ejection fraction, *TP* serum total protein

## Discussion

In a physiological state, pleural fluid enters the pleural space through the parietal pleura via a filtering pressure gradient and it is removed via an absorptive pressure gradient through the visceral pleura, together with lymphatic drainage through the stomas of the parietal pleura. Thus, filtration and reabsorption of pleural fluid maintain a dynamic balance through a negative feedback system between visceral and parietal pleurae [13, 14].

However, the physiological state is inevitably perturbed by the systemic response and local changes due to thoracic surgery. A systemic response is usually induced by anesthesia, surgical injuries, inflammation, and stress. Local changes including intrathoracic tissue injuries and changes such as residual lung over-inflation and alteration of the pulmonary circulation (blood re-distribution, the increase in blood flow, velocity, and hydrostatic pressure) can contribute to the development of pleural effusion [15–17].

Few previous studies aimed to assess the factors affecting PDV after thoracic surgery. Hristova et al. reported that age, chronic obstructive pulmonary disease (COPD), and lower lobectomy were risk factors for large drainage volume on the second day after lobectomy [18]. Kosugi et al. proved that reduced creatinine clearance and thoracic duct resection were risk factors for larger PDV after transthoracic esophagectomy [19]. However, these two studies included various factors that could affect the PDV, such as different surgical procedures, timing of operation, presence of pleural adhesion, and blood transfusion or complications intra- or postoperatively. In order to minimize the potential interference factors, all patients in the present study were analyzed based on the exclusion criteria. It may be an effective way to identify the predictive factors of PDV in patients who are diagnosed with NSCLC and who undergo uniportal VATS lobectomy.

Age is the most representative indicator of the physical condition, and our research showed that it was an independent predictive factor for PDV. This finding was similar to that in the study by Hristova et al [18]. The primary reason why PDV increases with age is increased microvascular dysfunction and hyperpermeability, which are caused by the destruction of endothelial cell adhesion and interstitial matrix-associated proteins [20].

Our research proved that FEV1% was another significant predictor of PDV. FEV1% is considered to be an effective indicator of bronchiole expansion function and pulmonary compliance, and reduction in FEV1% reflects the degree of alveolar hyperinflation and subsequent physiopathological decrease in the pulmonary vascular bed, which can aggravate postoperative microvascular filtration. A previous investigation on the operative patients also suggested a causative relationship between the decrease in compliance and the perturbation in pleuro-pulmonary fluid balance, which might lead to an increase in pleural effusion [16].

LVEF, an index of left ventricular systolic function, was also found to be a predictor of PDV. One possible explanation is that decreased left-sided cardiac function increased the hydrostatic pressure in the pulmonary circulation and pulmonary edema promoted pleural fluid filtration. In addition, secondary pulmonary edema could also aggravate the hypoxia and inflammation of the residual lung, which further increased the PDV [21–23].

The relationship between TP and pleural transudate has been adequately studied in previous research. Lower serum protein decreased the serum colloid osmotic pressure, which resulted in a higher PDV as a result of increased filtration and decreased reabsorption [14, 24, 25].

Patients who underwent lower lobectomy had higher PDV in our research, and this finding was in accordance with that reported by Hristova et al. and Kouritas et al. [18, 26, 27]. Increased PDV after lower lobectomy might be due to larger residual space after removal of the lower lobe than the upper or middle lobe, subsequently causing a greater reduction in the vascular bed and pleural reabsorption area as well as greater over-inflation and blood flow redistribution in the residual lung, which resulted in greater filtration of the pleural fluid.

In any case, the amount of pleural drainage cannot be ignored while judging the timepoint of chest tube removal. This study presents the important factors associated with PDV, which can help in perioperative patient management. These factors could be beneficial for alerting the surgeons of incremental PDV and for increasing the awareness. Before the operation, the patients with high predictive PDV should be administered an individualized correction through preoperative therapy and exercise for an enhanced recovery after surgery. After the operation, these predictors can help identify the patients who can safely undergo early chest tube removal after the operation and can provide evidence for differentiated and individualized chest tube management.

## Limitations

Although this study collected clinical data prospectively, it was a retrospective study. In addition, as the dependent variable (PDV) was continuous, we could not perform a logistic regression analysis to define the cut-off value for each independent variable. In our future study, we plan to define the cut-off value for each independent variable.

## Conclusions

It is possible to identify the predictive factors associated with an incremental risk of PDV after uniportal VATS lobectomy for NSCLC, and they can be used to tailor individualized safe chest tube management.

## Data Availability

The data used and/or analyzed during the current study were obtained from the Department of Thoracic Surgery, the First Hospital of Jilin University. The data are available from the corresponding author on reasonable request.

## Abbreviations

PDV: Pleural drainage volume
VATS: Video-assisted thoracic surgery
NSCLC: Non-small cell lung cancer
IQR: Interquartile range
BMI: Body mass index
FEV1%: Forced expiratory volume in 1 s % predicted
DLCO%: Carbon monoxide lung diffusion capacity % predicted
LVFS: Left ventricular fractional shortening
LVEF: Left ventricular ejection fraction
AST: Aspartate aminotransferase
ALT: Alanine aminotransferase
TP: Serum total protein
ALB: Serum albumin
CRE: Serum creatinine
BUN: Blood urea nitrogen
PT: Prothrombin time
APTT: Activated partial thromboplastin time
TT: Thrombin time
FIB: Fibrinogen
INR: International normalized ratio
RBC: Red blood cell count
WBC: White blood cell count
PLT: Platelet count
Hb: Hemoglobin
ESR: Erythrocyte sedimentation rate
TNM: Tumor, node, metastasis
LUL: Left upper lobe
LLL: Left lower lobe
RUL: Right upper lobe
RML: Right middle lobe
RLL: Right lower lobe
